# *From mine to apothecary*: an archaeo-biomedical approach to the study of the Greco-Roman lithotherapeutics industry

**DOI:** 10.1080/00438243.2018.1515034

**Published:** 2018-10-18

**Authors:** Effie Photos-Jones

**Affiliations:** a Analytical Services for Art and Archaeology (Ltd), Glasgow, UK; b Archaeology, School of Humanities, University of Glasgow, Glasgow, UK

**Keywords:** Minerals, microbiome, Greco-Roman lithotherapeutics, miltos, alum

## Abstract

Western biomedicine has only partially developed its own tradition of mineral medicinals (lithotherapeutics), at least compared to botanicals. This is perhaps because these minerals were site-specific, and fundamental information associated with the empirical processes of mineral extraction, beneficiation, storage, trade and preparation was not widely available. In other words, there are many and serious breaks in the multi-link chain from mine to apothecary. This long-term investigation aims to rebuild this chain, on a mineral-by-mineral basis, by pulling together the extant documentary record, material culture, mineralogy, geochemistry and microbial ecology, as well as by testing against known pathogens as an indicator of their antimicrobial activity. Critical to understanding the nature and efficacy of lithotherapeutics is the recognition that these materials need to be investigated simultaneously at two levels: the empirical (ancient sources and practices); and the biomedical (application of physical and biological sciences). Both approaches require the same starting point, namely the field (mine or quarry) and in particular the ‘point of contact’ (relationship) between minerals and their microbiome.

## About the minerals industry of antiquity

Lithotherapeutics or mineral medicinals have a small but conspicuous presence in the medical and scientific texts of the Greco-Roman (G-R) period, in, for example, Theophrastus (fourth-third centuries BC) (*On Stones*), Celsus (*De Medicina*) (first century AD), Dioscorides (*De Materia Medica*) (first century AD), Scribonius Largus (*Compositiones Medicamentum*) (first century AD), Pliny (*Natural History*) (first century AD) and Galen (second century AD) (many books). These had designated places of origin across the Mediterranean and, more often than not, multiple and diverse applications in the pigments industry, in medicine, agriculture, textiles and ship maintenance, to mention a few. Over the last few years, our work has focused on locating them in the field within their prescribed geographical regions and to study their nature and properties as iron oxides, layered aluminium silicates or aluminium sulphates (Hall and Photos-Jones 2008, 2009; Photos-Jones and Hall 2011, 2014; Photos-Jones et al. 2015, 2016, 2017, forthcoming).

**Figure 1.**
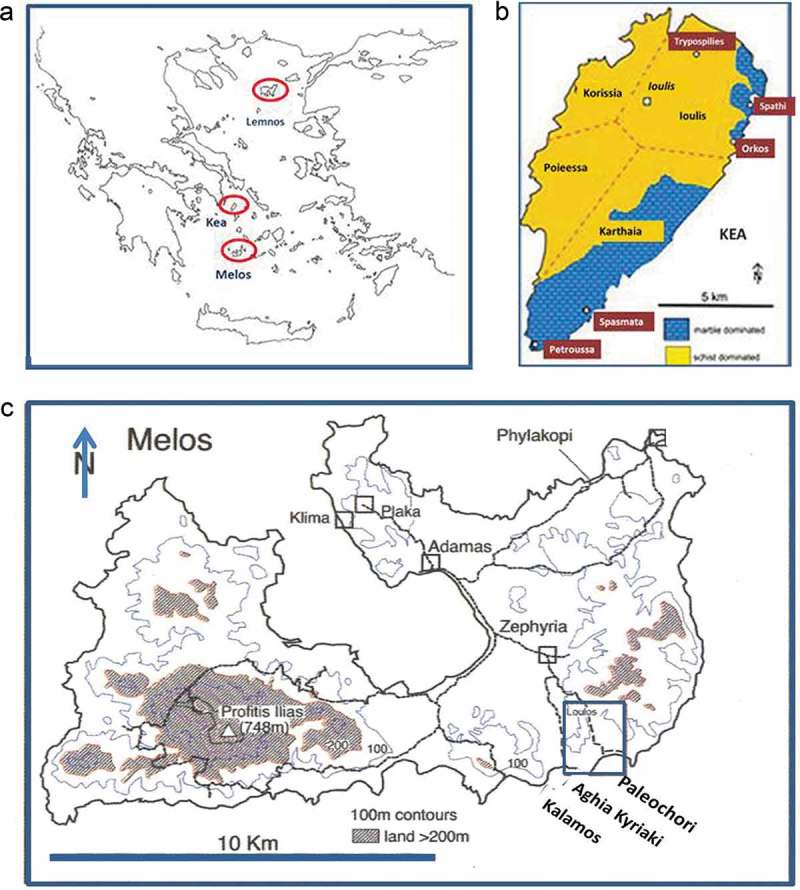
(a) Map of Greece and the Aegean Sea with the islands Melos, Kea and Lemnos highlighted. Samos is to the south of Lemnos; (b) Map of Kea, with localities of iron mines shown (adapted from Photos-Jones and Hall 2011, fig. 45); (c) Map of Melos showing the localities of Aghia Kyriaki and Palaeochori Bays with evidence for fumaroles and also archaeological remains dating from the Classical to Roman period (adapted from Photos-Jones and Hall 2014, fig 1.2b).

Their designated place of origin, be it a geographical (*Melian, Samian* or *Lemnian Earth* (Figure 1(a)) or type locality (e.g. the metal workers’ furnace or the potter’s kiln or a marine context) was a tacit way of recognizing that its source played a key role in defining its properties and by extension its markets. In the texts, the boundary between the raw mineral and the finished mineral product is often fluid and hard to delineate. There was certainly some type of treatment (grinding, washing, heating) which took place, sometimes, but not always, at source at the mine or the workshop (physical distances between the two rarely acknowledged) prior to packaging and shipment. It is not always clear why there was a processing stage in the first place: enrichment of the raw material, its separation into grades of different qualities, or simply manufacturing of new synthetic product(s) at the workshop are all possibilities. In general, there appears to have been no single overarching treatment that applied to all minerals, following their extraction.

For the metals-bearing minerals, it seems that the extractive industry worked in close proximity with metal manufacture, but industry boundaries (metal smelting/melting/forging) *vs* minerals processing are again not clearly delineated. The case of the mine of Soloi in the Troodos region of Cyprus visited by Galen in the late second century AD is an example of this lack of clear demarcation of activities between metal (copper) and mineral (copper/iron sulphates) extractive processes (Koucky and Steinberg 1987). Instead practices developed over time on a locality-by-locality and mineral-by-mineral basis, as demands for specific minerals ebbed and flowed across the near 1000-year span of the G-R world. It is almost certain that with our most technical and medical texts of G-R antiquity we only gain a very general picture of the range of activities carried out in each.

Amongst the G-R authors there were few individuals, one of them Galen, who realized the importance of assessing for oneself the difference between the extractive and the processing stages and for ensuring the procurement of the finished product, in its purest form and without the worry of adulteration as it travelled from mine to market and from apothecary to doctor. Galen appears to have gone to great lengths to visit Lemnos, Cyprus and Syria to witness processes first hand and to depart from these early ‘pharmaceuticals’ centres (Cyprus and Lemnos) with a supply of raw materials (*misy* and *chalkanthes* and *pompholyx* in Cyprus,^1^ and Lemnian Earths in the shape of a pellet or lozenge [20,000 of them] from Lemnos).^2^

Galen was right in being ‘curious’.^3^ Contrary to clays (for ceramics) and metalliferous ores (for metals), minerals as raw materials, as intermediate waste or as final products are often chemically and mineralogically indistinguishable from their surroundings, that is, the archaeological ‘soils’ into which they are embedded. This is because many, but by no means all, do not require high temperatures for their synthesis and purification and as such leave no distinct waste after their processing. Furthermore, upon exposure to the elements, synthetic minerals are likely to return to their stable mineral form, thus providing little archaeological evidence for their manufacture.

The lithotherapeutics of G-R antiquity formed only *one*, but a vital, sector of the industrial-minerals industry of antiquity, the other sectors being those dealing with pigments, mordants, cosmetics or washing powders (Photos-Jones and Hall 2011). It is not clear whether the procurement of *pharmaka* (drugs) was even perceived as a separate sector, rather that sectors may have overlapped. Ships certainly carried cargo that was meant for clients across many sectors of the economy (Morley 2007). Trade in minerals in general is better documented for the later periods, but later practices cannot be extrapolated back in time. For example, alum-containing amphorae of a particular type are known to have travelled around the Mediterranean in the medieval period carrying alum from a variety of sources (Jacoby 2005). This was potassium alum (alum-(K), KAl(SO_4_)_2_ · 12(H_2_O)) produced from the roasting, in kilns, of alunite (KAl_3_(SO_4_)_2_(OH)_6_) and its lixiviation (leaching out) as soluble material from the alunite rock (Singer 1948). For the Roman period it is the Richborough 527 amphora that is regarded as the type that carried alum around the Mediterranean. However, there is no direct evidence linking this type of amphora with alum, other than the fact that they originated from Lipari in the Aeolian Islands (Borgard 2005). Alum-(K) is very soluble in water and it is unlikely that any such containers will be found with their contents intact.

While soluble alum would have travelled in amphorae, other types of alum, like alum rock, did not. In the fifth century BC King Amassis of Egypt is said to have sent to Delphi 1000 talents of alum, equivalent to 26 tons (Herodotus *Histories* 2.180); this amount is unlikely to have travelled in ceramic containers.

Nigdelis’ (1990) account deriving from epigraphic evidence and associated with named *ergolavoi* (contractors) operating in the Hellenistic Cyclades in the Aegean Sea may throw some light on the identity and business interests of these ‘other’ individuals, but then again the evidence is at best scanty. *Ergolavoi* or *egonomoi* seem to have been primarily associated with the trade of industrial rocks, like marble from Paros, or sand and building stone from Mykonos, rather than minerals (Nigdelis 1990, 258). Around the second century BC, Italian *negotiatores* or men-in-business settled on Melos and by the Roman period Melos had a large community of these individuals (Alcock 1993). Some researchers thought that they were involved in the extraction and trade of industrial minerals (Pittinger 1975; Sparkes 1982). Again, they, as well as their trade, are elusive and their activities cannot be focused exclusively on minerals; they may have been trading in wheat or slaves.

The above brief summary alludes to the first stage of the journey, i.e. from mine to market port. The second leg of the journey, from port to apothecary, remains equally nebulous. Regarding some of the named professions involved in the industry, the *pharmakopolai* (sellers) and the *pharmakotrivai* (those who ground minerals in preparation for use), appear to have been involved in selling/working with *both* drugs *and* pigments. The *pharmakotrivai* seem to have belonged to the lower social strata and in the case of a certain Moschion in Demosthenes’ *Against Olympiodorus* (13–15), he must have been a slave. In the case of the *pharmakopolai*, as Totelin (2016a) has aptly demonstrated, although some of them appear to have been snake charmers or keepers of spiders and snakes, they are also presented in the sources as ‘taxpayers, trustees, and diplomats’, suggesting personal wealth (Totelin 2016a, 79). They were certainly sellers of *pharmaka*, as their name implies, but also seem to have dispensed them. One interesting note is the reference by Pseudo-Lucian that *‘pharmakopolai* keep their products in small boxes’ (Totelin 2016a, 80) which suggests that they may not have been wholesalers, but rather retailers as well as dispensers, the precursors to apothecaries of later periods. If that were indeed the case then the trajectory of *pharmaka*, whether as pigments or drugs, from source to market, must have been in the hands of some/many ‘invisible’ individuals, each passing their finished product to the next one in the chain that constituted the industry. But who these individuals were is difficult to ascertain. As ‘*mercatores’*, ‘*negotiatores’* or ‘*pragmatevomennoi’* they may have considered themselves as simply ‘engaging in business’.

Given the sparse and non-systematic information we have about the minerals industry of the G-R world deriving from the combined documentary and archaeological record, we have argued that there is a need for a fresh approach towards its study. This approach is two-pronged and consists of *an archaeological science component and a biomedical one*. Regarding the former, there is a need to shift the emphasis from the description of the mineral (often mineralogically vague and geochemically unknown) and its applications, i.e. the ailments it aimed to cure (often equally unclear) to the natural and cultural landscape of its origin. Although the scientific characterization of G-R minerals has been discussed extensively by many scholars, for example of Pliny (Bailey [1929] 1932; Levidis 1994; Healy 1999), of Dioscorides (Riddle 1985) or of Theophrastus (Eichholz 1965), their identity remains elusive because they lack materiality. When they do surface, as ingredients in medical boxes and from secure archaeological contexts like burials (Boyer et al. 1990; Ignatiadou 2015; Katsifas et al. 2018) or shipwrecks (Giachi et al. 2013) they can provide invaluable information about the recipes or the medicaments they were part of (collyria [eye ointments], plasters [wound dressings]). However, as ingredients in recipes they are one step removed form the orginal mineral. Further to that, taphonomic conditions may have imposed additional alterations.

The archaeological science component proposed here focuses on the elucidation of the G-R minerals’ natural and cultural landscape. The cultural landscape consists of both tangible (material culture) and intangible parameters (practices); the latter encompasses the means by which the mineral was extracted, processed, packaged, how it was shipped (in what type of containers and in what quantities), who managed its trade and distribution, and finally how it was dispensed: in short, the sum of empirical knowledge that underpinned the industry and on a mineral-by-mineral basis.

On the other hand, the biomedical component focuses on the investigation of the bioactivity of these minerals and their microbiome, individually and collectively, either as raw materials or as part of recipes. The aim of the combined approach is to translate empirical observations and practices in use for over two millennia into scientific language that could be meaningful today.

To illustrate this combined archaeo-biomedical approach, I focus on two minerals from two islands in the Aegean: the *miltos* of Kea and the *alum* of Melos (Figure 1(a-c)). *Miltos* (Theophrastus, *On Stones*, 52–4) was the prized red iron oxide (on its own or in association with calcite or clays) of antiquity, but beyond its property as a pigment and a cosmetic, it also had diverse applications in ship maintenance and agriculture (Lytle 2013; Photos-Jones et al. 1997, forthcoming). *Miltos* from other localities (Lemnos, NE Aegean and Cappadocia/Sinope, Turkey) was also known and it is the latter two that are usually presented as therapeutic (Dioscorides V. 96; Pliny 35.13). Theophrastus, the key source on Kea *miltos*, never claimed that Kea *miltos* was medicinal.

Alum had many and diverse applications as a mordant, a tanning agent, in metalworking and as a haemostatic. It is on the basis of its astringency that it was mainly identified. Astringency is a gustatory sensation (it was primarily a means of identifying the mineral in the field). Medically, an astringent is a ‘drug that causes cells to shrink by precipitating proteins from their surfaces’ (Oxford Concise Medical Dictionary, online). Although antiquity would have been unaware of this definition, it would almost certainly have had empirical knowledge thereof. Alum as alunite occurs in the vicinity of obsidian sources and once discovered, perhaps accidentally, it would have been used regularly by obsidian workers, in places like Melos in the Aegean or Lipari in the Tyrrhenian Sea, for minor cuts (Photos-Jones and Jones, forthcoming).

I will use the example of alum to make the case for the need to elucidate the cultural landscape, at source. Which mineral(s) qualified as Melian alum? Is there archaeological evidence for their extraction and processing? Which ones were bioactive?  I will use the example of Kea *miltos* to make the case for the need to understand the natural landscape, not simply in terms of its geochemistry and mineralogy, but also its microbial ecology. Could the minerals’ microbiome impart bioactivity over and above that provided by the mineral itself?

**Figure 2.**
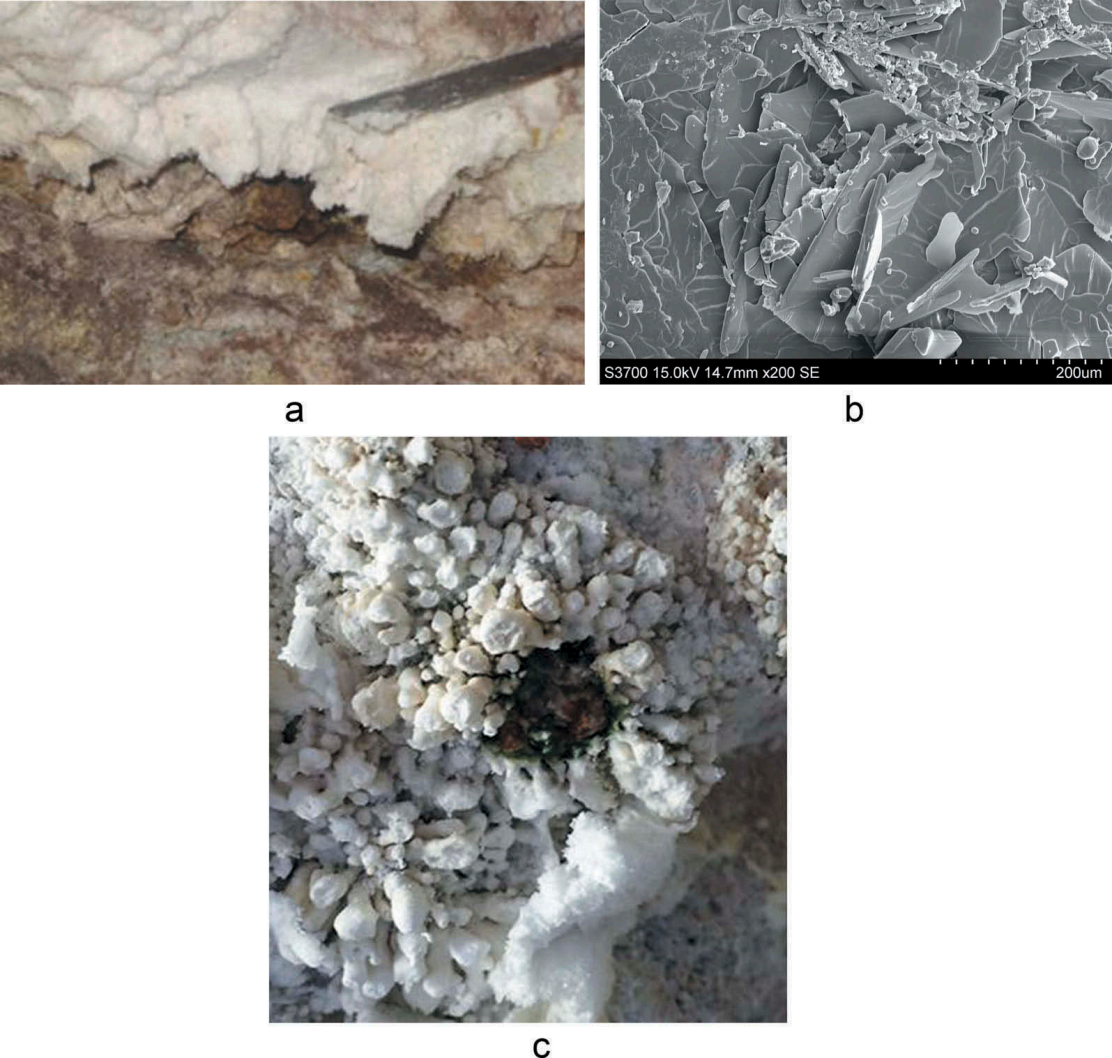
(a) ‘Slabs’ of renewable alunogen efflorescences forming within caverns in the area of the Kalamos promontory, SE Melos. Efflorescences can easily be removed with a knife (see blade) for scale; (b) SEM-EDAX of Melos alunogen with platy mineral habit. Scale on image bar = 2mm; (c) Microbial communities (dark green and moist) growing within well-defined boundaries and under hardened alum efflorescences. New, recently formed ones, and appearing ‘fluffy’, can be seen forming to the bottom left of the microbial colonies. Microbial growth seems to be more closely associated with older rather than new efflorescences. Diameter of area of microbial growth (green) = c. 2 cm.

Minerals do not exist in isolation but are often part of a biogeochemical cycle involving the presence and interaction with microbial communities in a two-way process (Haferburg and Kothe 2007). The microbial community is defined by its exposure to the conditions in the environment (temperature, pH), but these conditions can change by the activity of the microorganisms. Figure 2(c) shows a microbial community living in the warm and moist conditions associated with hydrothermal vents, Kalamos in SE Melos. The identity of micro-organisms, or the minerals’ microbiome (i.e. bacteria, fungi, algae, other), can be determined by DNA sequencing. A mineral’s bioactivity can potentially derive either from the mineral or its microbiome or both, since the latter may produce secondary metabolites (toxins) against specific pathogens.

In recent work we have attempted to provide some proof of bioactivity of some of these G-R minerals (Lemnian Earth, Samian Earth and alum), by investigating their antibacterial action. They were tested against specific pathogens, one gram negative (*pseudomonas aerugenosa*) and one gram positive (*streptococcus aureus*) bacterium (Photos-Jones et al. 2015, 2016, 2017). The results showed varying degrees of antibacterial action with the alum group mineral being the most effective (see Figure 4). Alum is a well-known antiseptic, so its antibacterial action is expected. What is new in this investigation is that a G-R source of alum is found to be strongly antibacterial.

**Figure 3.**
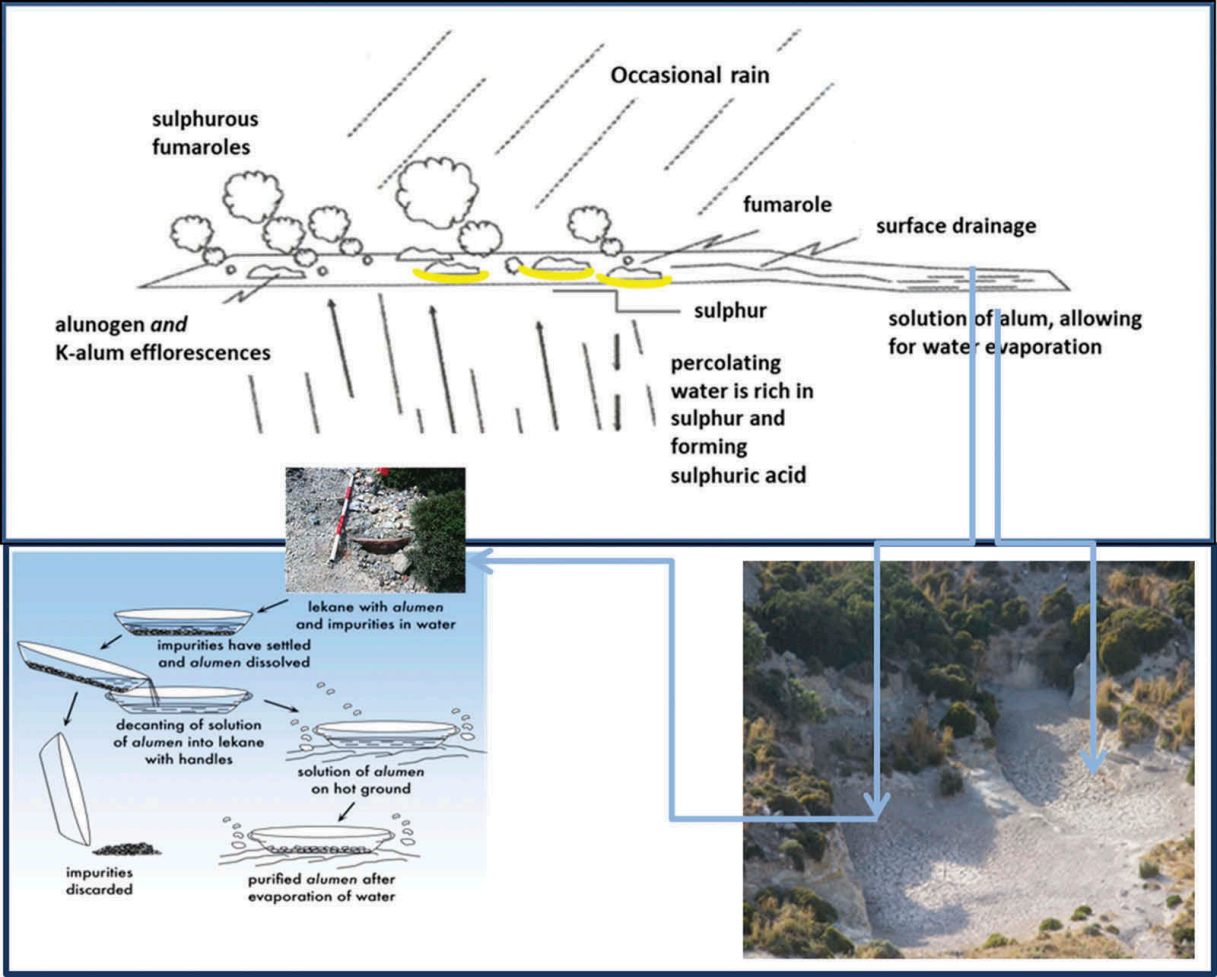
(a) (top) Sulphur-rich steam reaches the ground surface and solid sulphur is deposited near the fumarole entry. In the first rain sulphur reacts with water converting to sulphuric acid which percolates below ground dissolving the aluminium rich silicate rock. Aluminium is dissolved in the steam and when coming to the surface will precipitate as efflorescences around the mouth of the vent; (b) (bottom right) shallow basins. Similar structures could have been used for the evaporation of large quantities of solfataric alum; (c) (bottom left) schematic illustration of alum (alunogen and potassium alum) purification from a mixture of soluble and insoluble components of solfataric alum in open vessels *(lekanae)* (composite diagram adapted from Photos-Jones and Hall 2014).

**Figure 4.**
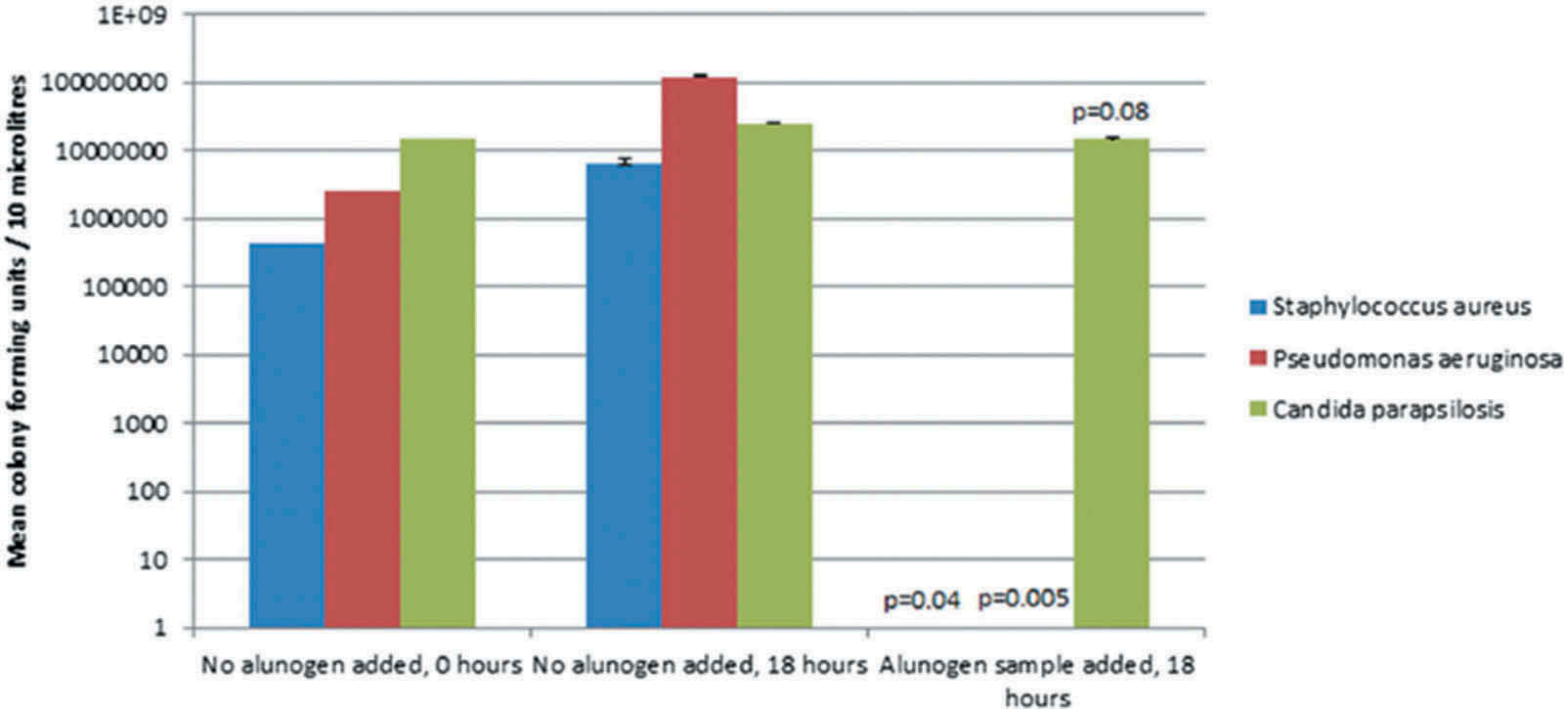
Alum (alunogen) of Melos: a strong antibacterial. A sample of experimentally processed alum is added to colonies of gram positive and gram negative bacteria; also of yeast. Eighteen hours after addition of the mineral, bacterial growth by either gram negative or gram positive bacteria was completely stifled. However the growth of yeast colonies (green bar) was not affected. (Diagram after Photos-Jones et al. 2016). The toxicity of processed Melian solfataric alum has not been tested.

## The alum of Melos: making the case for the cultural landscape

Melos has a geothermal field (ground temperature increases rapidly as a function of depth below surface) which is currently waning, but was, nevertheless, quite strong in the Classical and Roman period (Photos-Jones and Hall 2010). This is the result of past volcanic activity and is manifested in the form of fumaroles, hot springs on land and under water, and warm soils (*ca*. 60°C). Fumaroles are places (vents) where steam, containing gases like carbon monoxide and hydrogen sulphide, are emitted from depth, and a field of fumaroles is called a solfotara. On Melos, the majority of fumaroles occur today in the SE part of the island, around the two bays of Aghia Kyriaki and Palaeochori (Figure 1(c)). This is also the place with abundant evidence of settlements dating to Classical and Roman periods (Hall et al. 2003; Photos-Jones et al. 1999; Photos-Jones and Hall 2014). The material culture on these sites is associated with large deposits of pottery sherds deriving from *pithoi, lekanae* (shallow vessels) and *amphorae* embedded within sediments. The sheer size of the pottery-rich deposits, which remain to be fully investigated, points to large-scale processing of natural alum on site. The source of this alum must have been the promontory of Kalamos (Figure 1(c)) where there are still active fumaroles. Pliny’s account of what type of alum was available in Melos in the first century AD is given below.

Pliny’s view (*Natural History* 35.52, 183–5) was that

all alumen arises from water and slime, and is a natural exhudation from the earth. It is washed into a hollow in winter, and brought to maturity by the summer suns … the best quality is found in Egypt, the second best in Melos. The latter also is subdivided into liquid and solid alumen. The test for the liquid kind is that it should be clear and milky, not gritty when rubbed between the fingers, and with some spark of colour. This is called **phorimon** … . Liquid alumen has astringent, hardening and corrosive properties … . Another kind (the solid) is pale and rough …this they call **paraphoron**. (Natural History 35.52, 186): the best kind of all that which is called **melinum** from the island where it occurs … it surpasses the rest in its astringency … it is also the most compact variety … internal bleeding is arrested by a draught of alumen, and external bleeding by a rub of the same substance … (Natural History 35.52, 190): it should be understood that, in every case mentioned in connection with alumen from other sources, the variety from Melos is still more efficacious *…*(Bailey [1929] 1932).

On Melos there are two mineralogically distinct sources of alum: (a) alum from white rock containing alunite and (b) from sulphurous fumaroles, either in open-air solfataras or from within caverns. Figure 3(a), adapted from Hall et al. (2003), gives an illustration of how natural alunogen (Al_2_(SO_4_)_3_ · 17H_2_O) and/or alum-K (KAl(SO_4_)_2_ · 12 H_2_O efflorescences are formed around sulphur fumaroles. Steam rich in H_2_S comes to the surface and sulphur crystals form at the fumarole vent. In the first rain, sulphur dissolves in the water and forms sulphuric acid which seeps below the surface. The acid reacts with the rock below the surface, as temperature  rises rapidly, leaching out metals and giving the rock the white effect, characteristic of the island. Aluminium (cation) from the aluminium silicate within the volcanic rock reacts with the sulphate anion  in the steam and when it comes  up to the surface, it is precipitated as an alunogen efflorescence. The process repeats itself since alunogen is a renewable source, as long as Melos’ geothermal field is active. Alunogen is, therefore, a veritable ‘exudation of the earth’, as Pliny suggested.

Regarding the second phrase in his statement ‘it is washed into a hollow in winter, and brought to maturity by the summer suns*’*, alunogen efflorescences from open-air solfataras or those within caverns could have been be gathered within shallow basins carved into the rock. Figure 3(b) shows an image of two connecting basins (an area of 10 m x 10 m) carved into the bedrock in Kalamos, the function of which is yet to be fully explored. Alum efflorescences could have been collected within such large shallow basins which would have filled with rain water during the winter, the ponded water evaporating over the summer. At the end of the summer, pure alunogen would be collected and packaged in ceramic containers. The use of rain water for the dissolution of alum salts was essential because natural springs are rare in Melos (only two are reported) and the use of sea water would have been counterintuitive since it would have contaminated the alum solution, an already pure source, with ‘new’ salts, (NaCl, MgSO_4_). As such, water collection in basins or large pithos jars was the only alternative, and indeed large pithos were found buried in sediments at Aghia Kyriaki. For the evaporation of the water in the alum-rich solution, it is likely that,  both solar and geothermal (hot soils) energy would have been used (Photos-Jones and Hall 2014).

For smaller quantities of raw material or for a more regulated year-round industrial activity the schematic diagram in Figure 3(c) gives an illustration of how the alum purification process might have been carried out. The mineral would be placed in open vessels, the soluble alum salts (primarily alunogen and potassium alum) would go into solution with the insoluble constituents settling at the bottom and eventually discarded. The dissolved pure alum would be placed in another vessel and allowed to evaporate using solar and geothermal energy. In this way it would have been possible to carry out the process all year round and possibly under cover as well. The pure mineral would be packaged into amphorae, ready for transport.

In reference to the liquid variety called by Pliny *phorimon* (abundant), in an earlier publication, we have suggested that liquid alum may have been a supersaturated solution of aluminium sulphate minerals, the result of an intense Melos geothermal field, in the second century AD (Photos-Jones and Hall 2014). These conditions appear to have been very much in place from the Roman period (or earlier)  well into the eighteenth century giving rise to the reference to the ‘aluminous liquor’ seen by travellers to the island ‘distilling’ from the cavern walls (Tournefort 1718, 132). Today there are only very occasional seepages which is an indication that the geothermal field of Melos is waning. An alternatively hypothesis is that pure alunogen /potassium alum may have travelled not as a solid but as a supersaturated solution  (gel). Taking the original solution to complete dryness may have been cost ineffective as well as cause alterations in the hydration state of the desired minerals.

The second type of alum, distinct from solfataric alum, is alunite associated with kaolinite and quartz and occurring in Loulos, near Kalamos, in the SE of the island (Fig. 1c). This is likely to be the alum reported by Pliny as *melinum*. Large-scale exploration in the middle of the last century has obliterated much of the evidence. Loulos, which is contemporary with Aghia Kyriaki on the basis of similar pottery typology, is also the find location in the late nineteenth century of the Melos hoard, an impressive collection of silver coins dated to the fifth-century BC currently in the British Museum (Sheedy 2006). This suggests that the Loulos alunite deposit may have been  exploited in earlier periods.

Pliny’s (*Nat Hist* 35.52) account of Melian alum and its varieties makes sense in the framework of the geoarchaeological evidence. His ‘breathlessly’ swift and confident narrative of extraction, processing, trade and quality control, applications and prescriptions for ailments can be unpicked and systematically addressed. In summary, ‘the natural exudations’ from which *paraphoron* derived must refer to solfataric alum, while the *melinum* should refer to alunite in association with other minerals. The liquid *phorimon* (abundant) alum can either be equated with natural alum or with processed, packaged and exported in amphorae as gel; the *melinum* most likely refers to alunite in association with other minerals. Small blocks of Loulos *melinum* consisting of alunite, kaolinite and quartz, might have been most effective, as a haemostatic, having the ability to staunch blood flow by  narrowing blood vessels (acting as a vasoconstrictor) for small cuts and external applications. It is likely that this property was recognized, perhaps as early as the Neolithic and Early Bronze Age when Melos and Lipari were the major obsidian sources in the Mediterranean. In the Roman period they became the major producers of alum, the two raw materials – alum and obsidian – occurring in the vicinity of each other (on Melos at Nychia and Demenagaki– Figure 1(c)).

The principal and soluble component of Melos solfataric alum are the minerals alunogen Al_2_(SO_4_)_3_ · 17H_2_O and alum-(K) KAl(SO_4_)_2_ · 12 H_2_O forming as sheets within caverns from which they can be easily removed (Figure 2(a)). Alunogen has a hairy like structure, the scicssilis of Pliny, as can be seen in this Scanning Electron image (Figure 2(b))

To assess antibacterial activity of alunogen exploited in Melos during the Roman period we carried out microbiological testing of a sample consisting primarily of alunogen and potassium alum (Photos-Jones et al. 2016) (Figure 4). The sample reduced bacterial colonies by an order of 10^8^ against *Staphylococcus aureus* and an order of 10^7^ for *Pseudomonas aeruginosa*, which are gram positive and negative pathogens respectively. But there was no significant effect on the yeast *Candida parapsilosis*, showing that the alum can act as an antibacterial but not as an antifungal. In the natural alunogen of Melos, potentially processed in a simple dissolution-evaporation cycle, as outlined above and as hinted by Pliny’s relevant passage, the Greco-Roman world had a powerful antibacterial. But how toxic it was is not known.

## The miltos of Kea: examining a G-R mineral’s microbiome

There is one interesting inscription (IG II ^2^ 1128) found in the Agora of Athens in the late nineteenth century and dated to ca. 360 BC, which has long puzzled ancient historians (Lytle 2013). It contains a decree issued by Athens to the three city states of Kea (Ioulis, Korisseia and Karthaia (Ioulis, Korisseia and Karthaia (Figure 1(b))equiring Keians to export miltos from their respective mines in its entirety and exclusively to Athens, even bearing the charges for the transport. However, the iron oxide deposits at the silver mines at Laurion on the Attic mainland opposite Kea (Skarpelis and Argyraki 2009) could easily have provided Athens with sufficient (decorative) pigment. Therefore there must have been other reasons for the Athenian stern demand. Lytle (2013) has eloquently concluded that Kea miltos must have had other applications and in particular as an antifouling agent for boat maintenance, and in agriculture, as well as a pigment.

**Figure 5.**
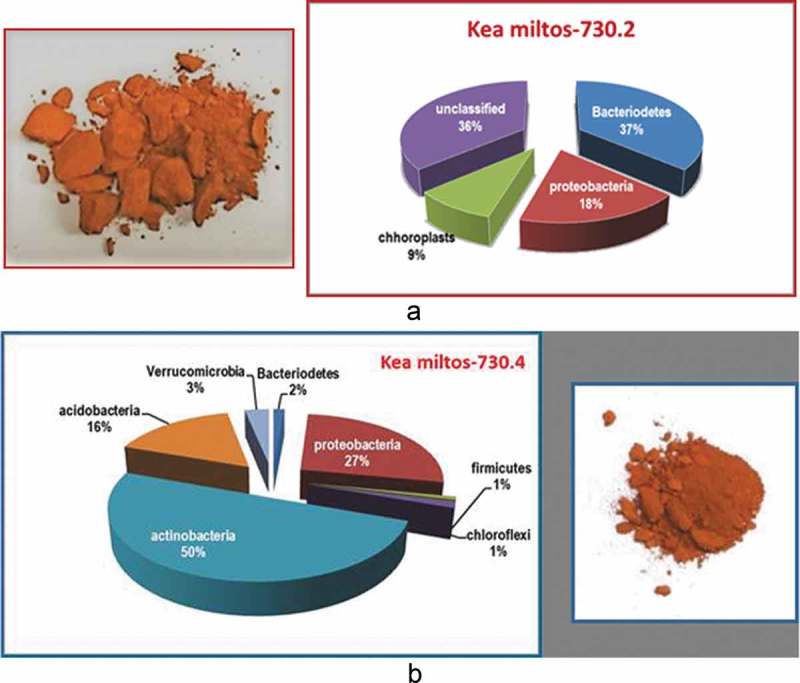
(a) Relative concentrations of microbial colonies associated with miltos sample 730.2 (adapted from Photos-Jones et al., 2018). There are only select microorganisms which can live in the toxic environment associated with miltos from the Pb-rich Petroussa mine; (b) Relative concentrations of microbial colonies associated with miltos sample 730.4 (adapted from Photos-Jones et al. in review).

The key properties that characterised characterized the *miltos* group were its colour (red) and its staining capacity (Figure 5(a,b)). It could be of pale, deep or medium red hue but always red (Theophrastus, *On Stones*, 52–4). Another property was its fine particle size (average *ca*. 150 nm) identified by Transmission Electron Microscopy (Photos-Jones et al., 2018). Theophrastus reports that *miltos* from Kea was extracted ‘*from the iron mines’*. The main ore bodies of Kea occur in the Petroussa area in the south, the Orkos area in the east and the Trypospilies area in the north of the island (Figure 1(b)). The dominant rock types on Kea are mainly calcareous chloritic schists and marbles. The mineralization consists mainly of Fe-oxides/oxyhydroxides ± galena, fluorite and barite, occurring at the zone of contact, filling cavities, between schists and marbles or within the marbles of the Blueschist Unit. There is no visible evidence for ore processing, but there is ample evidence for extraction from mines in the fifth and fourth centuries BC dated on the basis of pottery (Photos-Jones et al. 1997)

A small number of samples of Kea *miltos* had their microbiome fully characterized by DNA sequencing. For the purposes of this discussion, I present two samples (730.2 and 730.4, Figure 5(a,b)). 730.2, a pure iron oxide, consists of 82% goethite and 18% haematite. Sample 730.4, a mixture of iron oxides and calcite, consists of 52% goethite, 8% haematite, 7% quartz and 33% calcite. Both samples are rich in metallic (toxic) trace elements including zinc, arsenic and lead. More precisely, sample 730.4 contains 1000 ppm Mn, 330 ppm Zn, 800 ppm As, and 1100 ppm Sb; sample 730.2 contains 5700 ppm Pb, 46 ppm Mn, 470 ppm Zn, 2 ppm As, 80 ppm Sb (Photos-Jones et al., 2018). The high concentration of toxic elements particularly Pb (*ca* 6000 ppm), as in sample 730.2, would have made Kea miltos a good antifouling agent when applied onto a ship’s hull mixed with resin or pitch. It may be on account of high toxic-element concentration and natural fine particle size that Athens made sure that this vital supply came exclusively to them and in the quantities required for their fleet.

It should be noted here that the highly toxic Pb but also Fe and Cu are insoluble in water. This implies that although Pb concentration in the powdered sample 730.2 is *ca*. 5000 ppm, when the same sample is dissolved in water, Pb, Fe or Cu content are negligible (Photos-Jones et al., 2018). This means that *miltos* in an aqueous solution would not be toxic.

Turning now to the microbial ecology of these two samples, the amount of extractable DNA was sufficient for a phylogenetic characterization and furthermore for differentiation between samples. Sample 730.2 has primarily bacterial DNA with 10% of plant DNA (chloroplasts) (Figure 5(a)). It has a preponderence of bacteriodes (37%) and proteobacteria (18%) and a large number of unclassified microorganisms. By contrast, sample 730.4 has a much smaller amount of plant DNA (*ca*. 1%) and is mainly bacterial DNA and with a preponderence of actinobacteria (30%) and firmicutes (38%) (Figure 5(b)). The role of these bacteria is well known. For example, actinobacteria in the soil are saprophytes which live by breaking down organic matter and making it available for intake by plants. The same microorganisms are also prolific producers of secondary metabolites which are important as antibiotics (Ul-Hassan and Wellington 2009). Firmicute bacteria like *Paenibacillus* are growth promoters in plants (Bloemberg and Lugtenberg 2001) via phosphate solubilization, nitrogen fixation and disease control.

The above suggests that the bacteria living in the vicinity of miltos, *at source,* actually followed miltos at its destination, ie the farmer’s field or the ….shipyard.  ‘Arrival at destination’ is almost certain because most of the G-R lithotherapeutics were unlikely to have been processed beyond the grinding-dissolution-evaporation level and almost certainly were not heated at high temperatures. Application of effective (bioactive) miltos would have depended first on the empirical understanding of solubility properties of iron and other metal oxides/carbonates and second on making cause-effect deductions based on observation. Although G-R farmers would have been unaware of nitrogen fixing microorganisms (fermicutes) they would have likely noticed the beneficial effect of adding miltos to plant roots. In the absence of insoluble  iron, it would be the microorganisms that would drive the bioactivity rather than the mineral/ metallic elements.

## Conclusions

In this short paper I have tried to summarize our proposed two-pronged approach for the study of G-R lithotherapeutics: on the one hand, an archaeological-science aspect and on the other, a biomedical one. Our testing of G-R minerals for antibacterial properties against specific pathogens triggered the starting point of their investigation as therapeutics. Our hypothesis that these antibacterial properties may not originate only from the minerals themselves but from their microbiome as well, has formed the beginning of a long-term investigation.

There is a need to translate G-R minerals’ ‘vocabulary’, the set of empirical knowledge that is associated with their extraction, processing, packaging, trading and dispensing as constituents of *pharmaka*, into scientific terminology. In other words, it is on the G-R mineral *chaine operatoire* from mine to apothecary that we need to focus, rather than the G-R minerals, *per se*. For that we need to start at the beginning: the clearly stated places of their origin.

Regarding the identity of G-R minerals, we suggest that there cannot be a direct correspondence between individual G-R minerals and a modern mineral with a specific chemical formula and crystalline structure. Neither can it be said that G-R minerals were more akin to rocks, aggregates of minerals, since they could occur in near pure form as well. G-R minerals’ names could reflect group names or different grades thereof, or simply ‘commercial’ names. In other words, there are no steadfast rules as to how their name evolved. For those minerals which make their appearance in earlier, pre-Greek texts (for example tu-ru-pte-ri-ya for *stypteria* [*alumen*] and mi-to-we-sa for *miltos* in Linear B) it could, in principle, be traced in the meaning Bronze Age cultures gave to these materials. G-R minerals become real artefacts, or constituent parts thereof, albeit potentially much modified, only when retrieved from secure archaeological contexts as medicines within medicine boxes.

G-R lithotherapeutics were always thought to be specific to certain landscapes. It has been argued eloquently by Totelin (2016b) that in antiquity many (prized) materials had specific places of origin (i.e. Attic honey, Chian wine, Cypriot copper) and many places took pride in their products and the expertise that went with developing them. This is certainly true, but I suggest that for a minerals resource, in antiquity or today, it goes beyond a case of *appellation d’origine controlee*. The infrastructure required depending on the scale of production, the logistics of storage, packaging and shipment, the gradual evolution of technical knowhow, the scale and varying level of expertise of the workforce – all of these parameters represent levels of higher complexity than that associated with, for example, beekeeping or vineyard growing. As deduced by epigraphic record, the *negatiatores*, or men-in-business, may or may not have had a prominent role to play. Further, the toxic nature of the metallic minerals and the dire outcome of their unintended ‘misuse’ was well acknowledged by ancient authors (for example by Dioscorides V, 103 on *psimythion* [cerussa], the synthetic lead acetate (Stevenson 1955)). As such, minerals manufacture (as pigments, *pharmaka*, mordants or fertilizers) would have been perceived, at the societal level, as an ‘expert field’. However, we need to acknowledge, here, that the G-R sources are not particularly vocal on that front.

Finally, and in relation to medicinal earths, there has been a long-standing assumption (tacit *and* vocal) that they have acted largely as placebos rather than as real pharmacological agents. This was certainly the view of travellers, to the islands of their origin, in the post-medieval and later periods (Photos-Jones and Hall 2011). However, our own work has demonstrated their efficacy as antibacterials, and furthermore Williams and her colleagues have gone substantially further in providing a mechanism by which they have demonstrated that layered silicates in general can work as antibacterials (Morrison, Misra, and Williams 2016; Londono, Hartnett, and Williams 2017).

Turning to the biomedical aspect of this research, the proposed dual nature of *some* G-R minerals, as both mineral and microbiome, and with regards to the origin of their bioactivity, constitutes a new challenge. The proposal is based on the reading of the texts and the observation that the low-level processing that *some* G-R minerals underwent, (dissolution and precipitation at temperatures not above those needed for water evaporation), would have ensured that their microbiome (and their biomolecules) was carried into the final product, the mineral ingredient in the recipe. If some biomolecules produced by the microorganisms were bioactive, and many are known to be, then they would have contributed to the ‘minerals’’ overall bioactivity. Testing this hypothesis further, *and* proving it, is a much longer and complex endeavour. Nevertheless, I argue that having been perceived and/or proven to be efficacious for at least 2000 years, G-R minerals (and their microbiome) certainly deserve closer investigation both archaeologically and biomedically.

## Notes

1. Galen is thought to have visited the mine of Soloi, in the Troodos Mountain Range, NW Cyprus, in the late second century AD: ‘Because I wanted to do research in Cyprus about these medicines and because I had a friend who had a great deal of power on the island, who was also closely connected with the director of the mines and was the representative of Caesar [the emperor], I brought from there a lot of kadmeia and diphryges, spodion, pompholyx, and copper ore, and misy and sory and khalkanthos’ (Galen, *De Antidotis*, Kühn IV, p. 17), translated by Wallace and Orphanides (1990, 233). ‘For the same purpose [research] I went to Cyprus for its mines and to Coele Syria, which is part of Palestine, for the asphalt and other things there which are worthy of research. I did not hesitate to travel to Lemnos to see in what proportion blood was mixed with soil’ (Galen, *De Temp. Fac. Símp. Med* 9 (Kühn XII, p. 171) translated by Wallace and Orphanides (1990, 233).

2. Galen’s visit to Lemnos, what he saw and what he did, is described in great detail by the man himself. (Galen on Simple Drugs IX.II.247-8 and translated in Brock 1929, 194).

3. I am grateful to Dr Giorgos Constantinou, former director of the Geological Survey of Cyprus and researcher of Cyprus’ copper-extraction industry (Constantinou and Panagides 2013) for his insightful comment, namely that, in his view, ‘Galen was first and foremost a geologist . . . (and then a doctor).’

## References

[CIT0001] Alcock S. 1993 *Graecia Capta: The Landscapes of Roman Greece*. Cambridge: Cambridge University Press.

[CIT0002] Bailey K. C. [1929] 1932 *The Elder Pliny’s Chapters on Chemical Subjects. Parts I & II*. London: Arnold.

[CIT0003] Bloemberg G. V., and Lugtenberg B. J. J. 2001 “Molecular Basis of Plant Growth Promotion and Biocontrol by Rhizobacteria.” *Current Opinion Plant Biolology* 4: 343–350. doi:10.1016/S1369-5266(00)00183-7. 11418345

[CIT0004] Borgard P. 2005 “Les amphores à alun (Ier siècle avant J.-C.- IVer siècle après J.-C.)” In *L’Alun de Meditérranée. Colloque International, Naples, Lipari juin 2003*, edited by Borgard P., Brun J.-P., and Picon M., 157–170. Naples: Centre Jean Bérard.

[CIT0005] Boyer R., Barrandon J.-N., Binant C., Bui-Thi-Mai M., Girard M., Gratuze B., and Guineau B. 1990 “Les Collyres.” *Gallia* 47: 235–243. doi:10.3406/galia.1990.3167.

[CIT0006] Brock A. J. 1929 *Greek Medicine, Being Extracts Illustrative of Medical Writers from Hippocrates to Galen*. London: J M Dent and Sons.

[CIT0007] Constantinou G., and Panagides I. 2013 *Cyprus and Geology: Science, Environment, Culture*. Nicosia. In Greek. Nicosia: Bank of Cyprus Cultural Foundation.

[CIT0008] Eichholz D. E. 1965 *Theophrastus De Lapidibus. Edited with Introduction, Translation and Commentary*. Oxford: Clarendon Press, Oxford University Press.

[CIT0009] Giachi G., Pallecchi P., Romualdi A., Ribechini E., Lucejko J. J., Colombini M. P., and Mariotti Lippi M. 2013 “Ingredients of a 2000-Year-Old Medicine Revealed by Chemical, Mineralogical and Botanical Investigation.” *Proceedings of the National Academy of Sciences* 110 (4): 1193–1196. doi:10.1073/pnas.1216776110. PMC3557061 23297212

[CIT0010] Haferburg G., and Kothe E. 2007 “Microbes and Metals: Interactions in the Environment.” *Journal of Basic Microbiology* 47 (6): 453–467. doi:10.1002/(ISSN)1521-4028. 18072246

[CIT0011] Hall A., and Photos-Jones E. 2008 “Accessing past Beliefs and Practices: The Case of Lemnian Earth.” *Archaeometry* 50 (6): 1034–1049. doi:10.1111/j.1475-4754.2007.00377.x.

[CIT0012] Hall A., and Photos-Jones E. 2009 “The Juice of the Pomegranate: Quality Control for the Processing and Distribution of Alumen in Antiquity and Making Sense of Pliny’s *Phorimon* and *Paraphoron*” In *From Mine to Microscope: Advances in the Study of Ancient Technology*, edited by Shortland A. J., Freestone I., and Rehren T., 197–206. Oxford: Oxbow.

[CIT0013] Hall A. J., Photos-Jones E., McNulty A., Turner D., and McRobb A. 2003 “Geothermal Activity at the Archaeological Site of Aghia Kyriaki and Its Significance to Roman Industrial Mineral Exploitation.” *Geoarchaeology* 18 (3): 333–357. doi:10.1002/gea.10068.

[CIT0014] Healy J. F. 1999 *Pliny the Elder on Science and Technology*. Oxford: Oxford University Press.

[CIT0015] Ignatiadou D. 2015 “The Warrior Priest in Derveni Grave B was a Healer Too.” *Histoire, médecine et santé* 8: 89–113.

[CIT0016] Jacoby D. 2005 “Production et commerce de l’alun oriental en Meditérranée, XI^e^-XV^e^ siècles” In *L’Alun de Meditérranée. Colloque International, Naples, Lipari juin 2003*, edited by Borgard P., Brun J.-P., and Picon M., 219–268. Naples: Centre Jean Bérard.

[CIT0017] Katsifas C., Ignatiadou D., Zacharopoulou A., Kantiranis N., Karapanagiotis I., and Zachariadis G. 2018 “Non-Destructive X-Ray Spectrometric and Chromatographic Analysis of Ancient Macedonian Metal Containers and Their Contents.” *Separations* 5: 32. doi:10.3390/separations5020032.

[CIT0018] Koucky F. L., and Steinberg A. 1987 “Ancient Mining and Mineral Dressing on Cyprus” In *Early Pyrotechnology: The Evolution of the First Fire-Using Industries*, edited by Wertime T. A. and Wertime S. F., 149–180. Washington, DC: Smithsonian Institution Press.

[CIT0019] Levidis A. 1994 Πλίνιος ο Πρεσβύτερος, *Περί της Αρχαίας Ελληνικής Ζωγραφικής, 35ο βιβλίο της Φυσικής Ιστορίας (Pliny the Elder, on Ancient Greek Art Book 35 of Natural History)*. Athens: Agra Press (In Greek).

[CIT0020] Londono S. C., Hartnett H. E., and Williams L. 2017 “Antibacterial Activity of Aluminum in Clay from the Colombian Amazon.” *Environmental Science & Technology* 51 (4): 2401–2408. doi:10.1021/acs.est.6b04670. 28121138

[CIT0021] Lytle E. 2013 “Farmers into Sailors: Ship Maintenance, Greek Agriculture, and the Athenian Monopoly on Kean Ruddle (IG II2 1128).” *Greek, Roman, and Byzantine Studies* 53: 520–550.

[CIT0022] Morley N. 2007 *Trade in Classical Antiquity*. Cambridge: Cambridge University Press.

[CIT0023] Morrison K. D., Misra R., and Williams L. 2016 “Unearthing the Antibacterial Mechanism of Medicinal Clay: A Geochemical Approach to Combating Antibacterial Resistance.” *Nature Science Reports* 6: 19043. doi:10.1038/srep19043. PMC4705759 26743034

[CIT0024] Nigdelis P. M. 1990 “State and Society of the City-States of the Cyclades in the Hellenistic and Imperial Periods.” PhD diss., Aristotle University of Thessaloniki. In Greek with short English summary. doi:10.1099/00221287-136-2-327.

[CIT0025] Photos-Jones E., Cottier A., Hall A. J., and Mendoni L. G. 1997 “Kean Miltos: The Well-Known Iron Oxides of Antiquity.” *Annual British School at Athens* 92: 359–371. doi:10.1017/S0068245400016737.

[CIT0026] Photos-Jones E., and Hall A. J. 2010 “Stypteria Phorime as Alunogen in Solution: Possible Pointer to the Gradual Cooling of the Melos Geothermal System.” *Hellenic Journal Geosciences* 45: 117–126.

[CIT0027] Photos-Jones E., and Hall A. J. 2011 *Lemnian Earth and the Earths of the Aegean: An Archaeological Guide to Medicines, Pigments and Washing Powders*. Glasgow: Potingair Press.

[CIT0028] Photos-Jones E., and Hall A. J. 2014 *Eros, Mercator and the Cultural Landscape of Melos in Antiquity*. Glasgow: Potingair Press.

[CIT0029] Photos-Jones E., Hall A. J., Atkinson J. A., Tompsett G., Cottier A., and Sanders G. 1999 “The Aghia Kyriaki, Melos Survey: Prospecting for the Elusive Earths in the Roman Period in the Aegean.” *Annual British School at Athens* 94: 377–413. doi:10.1017/S0068245400000654.

[CIT0030] Photos-Jones E., Edwards C., Haener F., Lawton L., Keane C., Leonard A., and Perdikatsis V. 2017 “Archaeological Medicinal Earths as Antibacterial Agents: The Case of the Basel Lemnian Sphragides” In *Geology and Medicine: Historical Connections*, edited by Duffin C. J., Gardner-Thorpe C., and Moody R. T. J., 141–153. London: Geological Society Special Publication 452. doi:10.1144/SP452.6.

[CIT0031] Photos-Jones E., Keane C., Jones A. X., Stamatakis M., Robertson P., Hall A. J., and Leanord A. 2015 “Testing Dioscorides’ Medicinal Clays for Their Antibacterial Properties: The Case of Samian Earth.” *Journal of Archaeological Science* 57: 257–267. doi:10.1016/j.jas.2015.01.020.

[CIT0032] Photos-Jones E., Knapp C. W., Elgy C., Venieri D., Christides G., and Valsami-Jones E. 2018 “*Miltos*:The Nanosized, Antimicrobial Iron Oxides of Greco-Roman Antiquity.” *Journal of Archaeological Science: Reports*. 10.1016/j.jasrep.2018.07.017 PMC6360534 30775415

[CIT0033] Photos-Jones E., Christides G. E., Piochi M., Keane C., Mormone A., Balassone G., Perdikatsis V., and Leanord A. 2016 “Testing Greco-Roman Medicinal Minerals: The Case of Solfataric Alum.” *Journal of Archaeological Science: Reports* 10: 82–95. doi:10.1016/j.jasrep.2016.08.0.

[CIT0034] Photos-Jones E., and Jones R. E. forthcoming “Mycenaean ‘*tu-ru-pte-ri-ya’*: Alum, Vitriol or Simply an Astringent Mineral” In *Feschrift for Lucia Vagnetti*, Incunabula Graeca, edited by Bettelli M., Del Freo M., and van Wijngaarden G. J. Rome: Consiglio Nazionale delle Ricerche, Istituto di Studi sul Mediterraneo Antico.

[CIT0035] Pittinger J. 1975 “The Mineral Products of Melos in Antiquity and Their Identification.” *Annual British School at Athens* 70: 191–197. doi:10.1017/S0068245400006614.

[CIT0036] Riddle J. M. 1985 *Dioscorides on Medicine and Pharmacy*. Austin: University of Texas Press.

[CIT0037] Sheedy K. 2006 *The Archaic and Early Classical Coinage of the Cyclades*. London: Royal Numismatic Society Special Publications 40.

[CIT0038] Singer C. 1948 *The Earliest Chemical Industry: An Essay in the Historical Relations of Economics and Technology Illustrated from the Alum Trade*. London: Folio Society.

[CIT0039] Skarpelis N., and Argyraki A. 2009 “Geology and Origin of Supergene Ore at the Lavrion Pb-Ag-Zn Deposit, Attica, Greece.” *Resource Geology* 59 (1): 1–14. doi:10.1111/j.1751-3928.2008.00076.

[CIT0040] Sparkes B. A. 1982 “Production and Exchange in the Classical and Roman Periods” In *An Island Polity: The Archaeological of Exploitation in Melos*, edited by Renfrew C. and Wagstaff J. M., 228–235. Cambridge: Cambridge University Press.

[CIT0041] Stevenson L G. 1955 “On Tthe Meaning Oof The Words CerussaCERUSSA and PsimithiumPSIMITHIUM.” *Journal Oof The History Oof Medicine and Allied Sciences* 10 (1): 109-11. 10.1093/jhmas/x.1.109 13233507

[CIT0042] Totelin L. M. V. 2016a “*Pharmakopolai*: A Re-Evaluation of the Sources” In *Popular Medicine in Graeco-Roman Antiquity: Exploration*, edited by Harris W. V., 65–85. Leiden: Brill.

[CIT0043] Totelin L. M. V. 2016b “The World in a Pill. Local Specialties and Global Remedies in the Graeco-Roman World” In *The Routledge Handbook of Identity and the Environment in the Classical and Medieval Worlds*, edited by F. K. Rebecca and J. -L. Molly, 151–170. London: Routledge.

[CIT0044] Tournefort J. P. D. 1718 *A Voyage into the Levant*. London D. Browne, A. Bell, J. Darby et al. London: D.Browne.

[CIT0045] Ul-Hassan A., and Wellington E. M. 2009 “Actinobacteria” In *Encyclopedia of Microbiology*, edited by Schaechter M., 26–44. New York: Academic Press.

[CIT0046] Wallace P. M., and Orphanides A. G. 1990 *Sources for the History of Cyprus: Greek and Latin Texts to the Third Century A.D. Volume I*. Institute of Cypriot Studies, University of Albany, State University of New York and Cyprus College. Cyprus: Konos Press.

